# Neuroinflammation Associated With Inborn Errors of Immunity

**DOI:** 10.3389/fimmu.2021.827815

**Published:** 2022-01-19

**Authors:** Hannes Lindahl, Yenan T. Bryceson

**Affiliations:** ^1^ Clinical Immunology and Transfusion Medicine, Karolinska University Hospital, Stockholm, Sweden; ^2^ Center for Molecular Medicine, Department of Clinical Neuroscience, Karolinska Institute, Karolinska University Hospital, Stockholm, Sweden; ^3^ Center for Hematology and Regenerative Medicine, Department of Medicine, Karolinska Institute, Karolinska University Hospital Huddinge, Stockholm, Sweden; ^4^ Brogelmann Research Laboratory, Department of Clinical Sciences, University of Bergen, Bergen, Norway

**Keywords:** interferonopathies, primary immunodeficiencies, neuroinflammation, familial hemophagocytic lymphohistiocytosis (FHL), Mendelian genetic diseases, interleukin-1, type I interferon, autoinflammatory disorders

## Abstract

The advent of high-throughput sequencing has facilitated genotype-phenotype correlations in congenital diseases. This has provided molecular diagnosis and benefited patient management but has also revealed substantial phenotypic heterogeneity. Although distinct neuroinflammatory diseases are scarce among the several thousands of established congenital diseases, elements of neuroinflammation are increasingly recognized in a substantial proportion of inborn errors of immunity, where it may even dominate the clinical picture at initial presentation. Although each disease entity is rare, they collectively can constitute a significant proportion of neuropediatric patients in tertiary care and may occasionally also explain adult neurology patients. We focus this review on the signs and symptoms of neuroinflammation that have been reported in association with established pathogenic variants in immune genes and suggest the following subdivision based on proposed underlying mechanisms: autoinflammatory disorders, tolerance defects, and immunodeficiency disorders. The large group of autoinflammatory disorders is further subdivided into IL-1β-mediated disorders, NF-κB dysregulation, type I interferonopathies, and hemophagocytic syndromes. We delineate emerging pathogenic themes underlying neuroinflammation in monogenic diseases and describe the breadth of the clinical spectrum to support decisions to screen for a genetic diagnosis and encourage further research on a neglected phenomenon.

## Introduction

A monogenic disease may be suspected when onset is unusually early, there are similar manifestations in other family members, or there is consanguinity (1). With affordable high-throughput sequencing it is now feasible to pursue a genetic diagnosis in individual patients, which adds information on disease prognosis, potential treatments, and may also facilitate prenatal diagnostics.

The most well-recognized disease with a primary neuroinflammatory etiology is multiple sclerosis (MS). The pathogenesis is generally assumed to be autoimmune but the antigen specificity of the autoreactive lymphocytes that drive the disease remain undefined (2). Neuroinflammatory diseases for which an autoimmune pathogenesis is more substantiated include neuromyelitis optica (NMO) spectrum disorder and autoimmune encephalitis. Conversely, other forms of neuroinflammation appear less likely to be driven by autoreactive lymphocytes but rather dysregulated innate immune cells, such as those observed in Bechet’s disease, an idiopathic systemic vasculitis syndrome (3). Moreover, neuroinflammation can cause collateral damage in central nervous system (CNS) infection, neurodegeneration, ischemia, and trauma.

**Figure 1:**
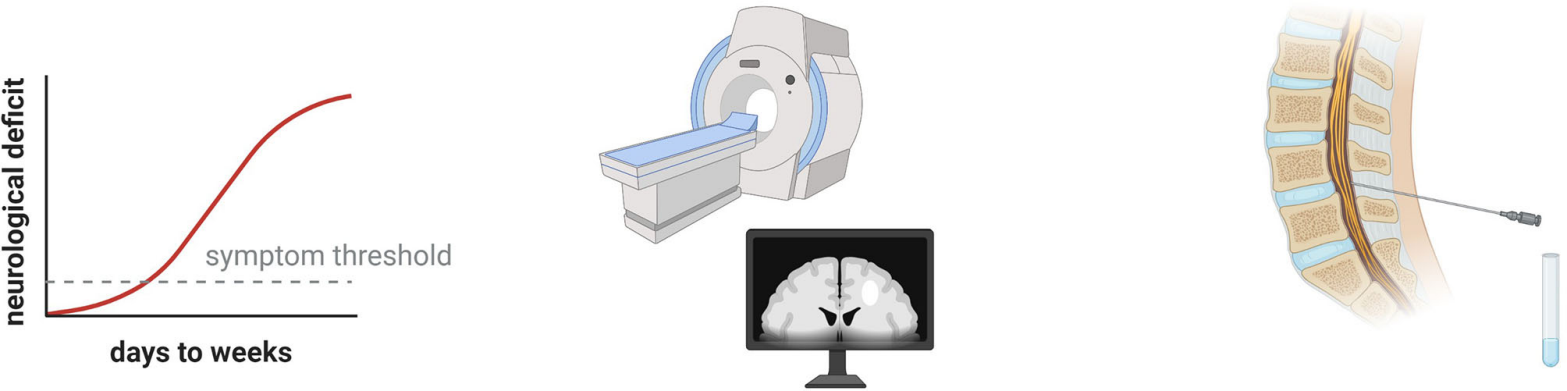
Clinical hallmarks of neuroinflammation. The patient may have a subacute onset of neurological symptoms that may resolve spontaneously or respond to immunomodulatory drugs. Using magnetic resonance imaging (MRI), acute lesions with blood brain barrier disruption can be visualized as contrast enhancing T1 lesions. Various pathologies can appear as T2 lesions, such as demyelination, axonal damage, and edema. Analysis of cerebrospinal fluid allows quantitative and qualitative assessment of intrathecal inflammation as well as demonstration of blood brain barrier disruption.

In clinical practice, it is not uncommon that a patient presents with apparent neuroinflammation, *e.g.* with subacute onset of CNS symptoms associated with abnormalities on magnetic resonance imaging (MRI) and cerebrospinal fluid (CSF) analysis (**Figure 1**), but without distinct features that justify a definite diagnosis. The patient will then typically be managed under a presumptive diagnosis until the clinical picture develops further, which often entails accumulation of neurological deficits. A prompt molecular diagnosis can thus benefit treatment. Early clinical presentation and a history consistent with a hereditary disease may suggest a genetic etiology, warranting genetic investigations. Furthermore, the increasing affordability implies that genome sequencing may soon constitute part of the routine first-line diagnostic work-up of severe neuroinflammation. Recently, McCreary and colleagues published an extensive panel of neuroinflammation-associated genes and demonstrated that it could provide a molecular diagnosis in 20% of unresolved pediatric patients with suspected genetic neuroinflammation (4). Herein, we review the literature on neuroinflammatory manifestations associated with monogenic immune-mediated diseases and discuss the different underlying immunological mechanisms. Our compilation can concretize what may constitute a monogenic neuroinflammatory clinical presentation.

## Autoinflammatory Disorders

Autoinflammatory disorders are clinical disorders of the innate immune system characterized by chronic or periodic inflammation without engagement of adaptive lymphocytes, *i.e.* autoreactive antibodies or T cells. The most recognized monogenic autoinflammatory syndromes are familial Mediterranean fever (FMF), cryopyrin-associated periodic syndrome (CAPS), hyperimmunoglobulinemia D syndrome (HIDS), and tumor necrosis factor receptor-associated periodic syndrome (TRAPS). IL-1β-driven or -biased inflammation is a common feature of these four syndromes. In disorders caused by NF-κB dysregulation the uncontrolled production of cytokines involves IL-1β as well as several other proinflammatory cytokines. By comparison, interferonopathies represent a distinct category of rare diseases characterized by increased type I IFN signaling and loss of negative regulation. And lastly, although caused by impaired lymphocyte effector functions, the most prominent feature of hemophagocytic syndromes is hyperinflammation with uncontrolled innate immune activation and they are therefore included under this heading in this review.

### IL-1β-Mediated Autoinflammatory Disorders

#### Familial Mediterranean Fever

Familial Mediterranean fever (FMF; MIM: 249100) is classically regarded as an autosomal recessive disease caused by variants in *MEFV*, but one mutant allele is sufficient to cause subclinical systemic inflammation (5) (**Table 1**). Furthermore, autosomal dominant FMF has also been described (MIM: 134610) (55) and likely involves a distinct subset of *MEFV* variants (56). *MEFV* encodes pyrin, an intracellular pattern recognition receptor that is expressed mainly by neutrophils and monocytes/macrophages. Upon its activation, an inflammasome complex forms, leading to proteolytic maturation of IL-1β as well as IL-18 and their subsequent extracellular release through a form of programmed cell death called pyroptosis (57). *MEFV* variants that cause FMF represent pyrin gain-of-function variants that lower the activation threshold of inflammasome formation. Clinically, FMF is characterized by periodic fever, serositis, arthralgia, and rash. A feared complication is renal failure due to amyloidosis. Apart from headache (10%) and seizures (4%), other neurological manifestations of FMF are very rare (8). Recurrent aseptic meningitis in FMF is occasionally reported (58–61), as well as a wide range of other neuroinflammatory manifestations, however the causal relationship with FMF is uncertain (8). Moreover, elevated protein in CSF as a sign of meningeal irritation may occur without clinical evidence of meningitis (6). Whereas *MEFV* variants that are associated with FMF are gain-of-function variants, a subset of rare *MEFV* variants that result in defective binding to an inhibitory 14-3-3 protein cause a distinct autosomal dominant disease termed pyrin-associated autoinflammation with neutrophilic dermatosis (PAAND), for which neurological involvement has not been reported (62, 63). Collectively, awareness that the episodic inflammation in FMF may also engage the CNS is important.

**Table 1:** Summary of neuroinflammatory manifestations associated with inborn errors of immunity.

Disease	Gene	Mutation type	Mode of inheritance	Immunopathology	Typical presentation	Reported neurological manifestations (estimated frequency)
Familial Mediterranean fever (FMF)	*MEFV*	Likely GoF	AR/AD	Lowered threshold of pyrin inflammasome activation leads to excessive IL-1β release.	Episodic (1-3 days) fever, serositis, arthralgia, and rash. Onset in childhood typically.	Headache [>10% (6)], seizures [4% (7)], Aseptic meningitis [<1/1000 (8)] as well as sporadic reports of demyelination (9), CNS vasculitis (10, 11), and optic neuritis (12).
Hyper-IgD syndrome (HIDS)	*MVK*	LoF	AR	Mevalonate kinase deficiency leads to deficiency of cholesterol derivatives, which results in inflammasome activation and excessive IL-1β release.	Episodic (3-7 days) fever, enlarged secondary lymphoid organs, and rash. Onset in childhood typically.	Headache [10-40% (13, 14)] and seizures. Sporadic reports of aseptic meningitis (13), cerebellar syndrome (13), and transverse myelitis (14).
Cryopyrin-associated periodic syndrome (CAPS)	*NLRP3*	GoF/LoF	AD	Lowered threshold of NLRP3 inflammasome activation leads to excessive IL-1β release.	Episodic fever, myalgia, and urticarial rash. Wide spectrum of severity and age of onset depending on type the genetic variant.	Headache [30-80% (15, 16)], sensorineural hearing loss [40-70% (15, 16)], papilledema [30% (15, 16)], and aseptic meningitis [5% (15)]. Sporadic reports of white matter lesions (15).
NLRP12-linked autoinflammatory disease (NLRP12-AID)	*NLRP12*	GoF/LoF	AD	Lowered threshold of NLRP12 inflammasome activation leads to excess IL-1β.	Familial cold autoinflammatory syndrome – cold induced episodes (1-3 days) of urticarial rash, fever, and arthralgia.	Transient sensorineural hearing loss (10%) (17) and sporadic reports of optic neuritis (18).
Tumor necrosis factor receptor-associated periodic syndrome (TRAPS)	*TNFRSF1A*	GoF	AD	Impaired signaling *via* the TNF-receptor results in enhanced secretion of IL-1, IL-6, and TNF.	Episodes (5-21 days) of fever, abdominal pain, and rash.	Headache (23%), seizures (1%), and vertigo (1%) (19). Sporadic reports of diplopia (17), cerebrovascular lesions (17), paresthesia (20), CSF pleocytosis (20), white matter lesions (20),and Tolosa-Hunt syndrome (21).
A20 haploinsufficiency	*TNFAIP3*	LoF	AD	Loss-of-function variants in A20, an inhibitor of the NF-κB signaling pathway, leads to excessive expression of pro-inflammatory cytokines.	Mucosal and cutaneous lesions, gastrointestinal symptoms, and episodic fever. Onset typically in early childhood and occasionally later, up to early adulthood.	Sporadically reported neuropsychiatric SLE with headache, seizures, cognitive impairment, ptosis, difficulty with upward gaze (22), CNS vasculitis (23), and aseptic meningitis (24).
Aicardi-Goutières syndrome (AGS) 1	*TREX1*	LoF	AR/AD	Defective processing of and sensing of nucleic acids results in immune activation and excessive type I interferon production.	Early onset encephalopathy associated with intracranial calcifications, leukoencephalopathy, cerebral atrophy, CSF pleocytosis, and cutaneous manifestations.	Apart from the typical presentation, sporadically reported encephalitis (25) and bilateral striatal necrosis (26).
AGS2	*RNASEH2B*	LoF	AR			
AGS3	*RNASEH2C*	LoF	AR			
AGS4	*RNASEH2A*	LoF	AR			
AGS5	*SAMHD1*	LoF	AR			
AGS6	*ADAR*	LoF	AR			
AGS7	*IFIH1*	GoF	AD			
AGS8	*LSM11*	LoF	AR			
AGS9	*RNU7-1*	LoF	AR			
Pseudo-TORCH syndrome 2	*USP18*	LoF	AR	An exaggerated response to normal levels of type I interferon leads to immune activation.	Microcephaly, hydrocephalus, cerebral calcification, systemic sterile inflammation at birth resembling a congenital infection, and cerebral hemorrhage.	Included in typical presentation
Pseudo-TORCH syndrome 3	*STAT2*	GoF	AR			
Retinovasculopathy and cerebralleukodystrophy with systemic features (RVCLS)	*TREX1*	GoF	AD	TREX1 variants with retained exonuclease activity but altered cellular localization lead to retinal and small vessel cerebral vasculopathy.	Early onset cerebrovascular disease, visual impairment, and occasionally involvement of other organs.	White matter lesions (92%), cerebral calcifications (52%), focal neurologic defects (56%), migraine (53%), cognitive impairment (47%), psychiatric disturbances (39%), and seizures (14%) (27).
ISG15 deficiency	*ISG15*	LoF	AR	ISG15 deficiency leads to increased type I interferon and decreased type II interferon signaling.	Moderately severe interferonopathy with cerebral calcifications and increased susceptibility to mycobacterial infections.	Cerebral calcifications (nearly 100%) and sporadic reports of seizures (28).
DNase II deficiency	*DNASE2*	LoF	AR	Loss of DNase endonuclease activity leads to aberrant sensing of self-DNA and elevated type I interferon induction.	Neonatal anemia, glomerulonephritis, liver fibrosis, deforming arthropathy, and increased anti-DNA antibodies.	Sporadic reports of headache, cerebral calcifications, white matter lesions, and learning difficulties (29).
Spondyloenchondrodysplasia (SPENCD)	*ACP5*	LoF	AR	Deficiency of ACP5, which may be a negative regulator of type I IFN, results in excessive type I interferon signaling.	Bone lesions and short stature, neurological manifestations, immune manifestations such as autoimmune thrombocytopenia, SLE, and vasculitis.	Cerebral calcifications (62%), spastic paresis (44%), and developmental delay (28%). Occasionally ataxia, seizures, psychosis, and neuropathy (30).
Chronic atypical neutrophilic dermatosis with lipodystrophy and elevated temperature (CANDLE).	*PSMB8*	LoF	AR	Plausibly immunoproteosome dysfunction leading to accumulation of damaged proteins resulting in cellular stress and type I interferon induction.	Onset during first year of life of recurrent fever, rashes, arthralgia, and progressive lipodystrophy.	Sporadic reports of cerebral calcifications and aseptic meningitis (31).
Familial HLH 2	*PRF1*	LoF	AR	Defects in genes involved in lymphocyte cytotoxicity lead to aberrant activation of macrophages and T cells resulting in excessive cytokine secretion.	High fever and life-threatening sepsis-like disease, often preceded by an immunological trigger event.	Pathological brain MRI or signs of inflammation in CSF (33-91%) (32). Occasionally ataxia, seizures, headache, motor impairment, and visual abnormalities (33).
Familial HLH 3	*UNC13D*	LoF	AR			
Familial HLH 4	*STX11*	LoF	AR			
Familial HLH 5	*STXBP2*	LoF	AR			
Griscelli syndrome, type 2	*RAB27A*	LoF	AR	Deficiency impairs cytotoxic lymphocyte granule docking and exocytosis.	Hypopigmentation, susceptibility to bacterial infections, and cytopenias. Predisposition for HLH.	Secondary to HLH: Sporadic reports of seizures (34, 35), cerebellar symptoms (36), elevated protein and cells in CSF, white matter lesions, motor impairment (37), encephalopathy (38, 39), and seizures (40).
X-linked lymphoproliferative syndrome (XLP) type 1	*SH2D1A*	LoF	XLR	SAP deficiency inhibits signaling for lymphocyte interactions, specifically impairing control of EBV infected B cells by CD8^+^ T cells and NK cells.	Hypogammaglobulinemia, B cell lymphoma, and HLH. Onset is almost always triggered by an EBV-infection.	Sporadic reports of CNS vasculitis manifested as memory deficit, motor impairment, headache, seizures, lesions on MRI, and pathological CSF (41–45).
Immunodysregulation polyendocrinopathy enteropathy X-linked (IPEX)	*FOXP3*	LoF	XLR	Tolerance defect caused by impaired development of Treg cells.	Onset in infancy of enteropathy, dermatitis, and autoimmunity such as insulin-dependent diabetes mellitus, hypoparathyroidism, and autoimmune cytopenias.	Seizures (14%), ventriculomegaly (14%), and developmental delay (3%) (46).
Autoimmune lymphoproliferative syndrome (ALPS) type IA	*FAS*	LoF	AD	Defective apoptosis of thymic lymphocytes resulting in non-malignant lymphoproliferation and autoimmunity.	Lymphadenopathy, splenomegaly, and autoimmune cytopenias. Onset typically during childhood but occasionally later.	Sporadic reports of cerebellar lesions, spinal degeneration, neuromyelitis optica, and Guillain-Barré syndrome (47–50).
ALPS type IB	*FASLG*	LoF	AD			
ALPS type II	*CASP10*	LoF	AD			
C1q-deficiency	*C1QA, C1QB, C1QC*	LoF	AR	Deficiency in C1q leads to defective clearance of apoptotic cells and immune complexes resulting in immune activation and autoimmunity.	skin lesions, chronic infections, increased susceptibility to bacterial meningitis, and autoimmune diseases (particularly SLE).	Neuropsychiatric lupus (50%) and seizures (12%) (51). Sporadic reports of cerebral vasculitis with motor impairment, encephalopathy, tremor, infarcts, transverse myelitis (51), cerebral calcifications (52), and pathological CSF (53).
Mendelian predisposition to herpes simplex encephalitis	*TLR3, UNC93B1, TRIF, TRAF3, TBK1, IRF3, NEMO, IFNAR1, STAT1*	LoF	AR, AD, XLR	Impaired recognition of double-stranded viral DNA by TLR3 or impaired type I interferon receptor signaling.	Susceptibility to herpes simplex virus 1 encephalitis. IFNAR1 and STAT1 defects also result in susceptibility to mycobacterial infections.	Included in typical presentation
GATA2-deficiency	*GATA2*	LoF	AD	Defective stem/progenitor cell renewal and differentiation, leading to monocyte, B cell, and NK cell deficiency.	Cytopenias, susceptibility to infections, and MDS/AML.	Sporadic reports of intellectual disability, transient ischemic cerebral palsy, and progressive multifocal leukoencephalopathy (PML) (54).
RANBP2-deficiency	*RANBP2*	LoF	AD	Acute necrotizing encephalopathy (ANE) may occur in otherwise healthy children after common viral infections. The mechanistic link between RANBP2 and ANE is not known.	Multifocal symmetric brain lesions, focal neurologic symptoms, and seizures.	Included in typical presentation.

GoF, gain of function; LoF, loss of function; AR, autosomal recessive; AD, autosomal dominant; XLR, X-linked recessive.

#### Mevalonate Kinase Deficiency

Mevalonate kinase deficiency (MKD) consists of the autosomal recessive syndromes mevalonic aciduria (MVA; MIM: 610377) and hyperimmunoglobulinemia D syndrome (HIDS; MIM: 260920). Both are caused by loss-of-function variants in *MVK*, which encodes the cholesterol biosynthesis enzyme mevalonate kinase. MVA and HIDS represent opposite ends of a clinical spectrum associated with absent to subnormal enzyme activity, respectively (64). The exact pathogenesis is elusive but involves build-up of mevalonate and loss of pyrin inhibition resulting in inflammasome activation and excess IL-1β production (65). The mevalonate pathway produces the substrate for a form of post-translational modification, prenylation, which is involved in the regulation of TLR-induced phosphoinositide 3-kinase (PI3K) activation (66). Furthermore, impaired prenylation of RhoA, a small GTPase, inactivates RhoA, decreases pyrin phosphorylation and subsequent 14-3-3-mediated negative regulation of pyrin activity (67). Loss of prenylation in MKD thus contributes to pyrin activation and the hyperinflammatory phenotype. MVA is associated with severe developmental delay, ataxia, epilepsy, and shortened lifespan (65). HIDS is an autoinflammatory periodic fever syndrome associated with persistently elevated IgD and increased mevalonic acid in the urine during attacks (13). Common clinical manifestations of HIDS include rash, hepatosplenomegaly, and lymphadenopathy. The most common neurological manifestation is headache (10-40%), which may be present independent of the fever episodes (65). Sporadic reports of seizures, transverse myelitis, cerebellar syndrome, and aseptic meningitis exist (13, 14). Although the exact pathogenesis of FMF and HIDS are not completely understood, similarities in the affected pathways are consistent with a similar pattern of CNS engagement that mainly involves headache and only rarely overt neuroinflammation.

#### Cryopyrin-Associated Periodic Syndrome

Cryopyrin-associated periodic syndrome (CAPS) can be regarded as a continuum of three phenotypes that were described before the common disease-causing gene was identified – from the milder familial cold autoinflammatory syndrome (FCAS, MIM: 120100), *via* Muckle-Wells syndrome (MWS, MIM: 191900), to the most severe form chronic infantile neurological cutaneous and articular syndrome (CINCA, MIM: 607115). All three are caused by autosomal dominant variants in *NLRP3 (* 68–70). Analogous to *MEFV*, *NLRP3* encodes an intracellular pattern recognition receptor, cryopyrin, and the CAPS-associated gain-of-function variants lower the threshold of inflammasome activation leading to excessive IL-1β production and release. Additionally, NLRP3 acts as a negative regulator of NF-κB signaling and loss-of-function *NLRP3* variants are also reported as the cause of autoinflammation (71). Clinically, CAPS is characterized by periodic fever, urticarial rash, conjunctivitis, and arthralgia. Neurological symptoms are common and most frequently consist of headache (30-80%), sensorineural hearing loss (40-70%), and papilledema (30%) (15, 72, 73). Less common neurological manifestations include aseptic meningitis (5%) and hydrocephalus, seizures, chorea, white matter lesions, and CSF pleocytosis have sporadically been reported (16, 74–76). A case has been described that mimicked Tolosa-Hunt syndrome with steroid-responsive periorbital pain, diplopia, and granulomatous inflammation (77). Neuroradiological manifestations are not well characterized, but a case report in which serial imaging was performed showed enhancement in leptomeninges, cochlea, and cranial nerves (78). The higher frequency of neurological symptoms in CAPS compared to the other IL-1β-mediated autoinflammatory disorders may be linked to the increasingly appreciated role of NLRP3 in microglia as the main contributor to neuroinflammation in neurodegeneration, infection, and stroke (79).

#### TNF Receptor-Associated Periodic Syndrome

TNF Receptor-Associated Periodic Syndrome (TRAPS; MIM: 142680) is an autosomal dominant periodic fever syndrome that is caused by gain-of-function variants in *TNFRSF1A*, which encodes one of the major TNF receptors (80). Despite that variants associated with TRAPS impair signaling *via* the TNF receptor, inflammation is enhanced with increased IL-1β, IL-6, and TNF secretion, likely due to intracellular accumulation of misfolded protein (81). Approximately 10% of cases have adult onset. The clinical presentation typically consists of fever, abdominal pain, arthralgia, myalgia, and a migratory rash. Neurological symptoms are not core features of TRAPS, but approximately 20% of patients manifest headache and to lesser extent seizures (1%), vertigo (1%), diplopia, and cerebrovascular lesions (17, 19, 21). Occasionally paresthesia, CSF pleocytosis, white matter lesions (20), and Tolosa-Hunt syndrome have been reported (21).

#### Other IL-1β-Mediated Autoinflammatory Disorders

Variants in *NLRP12* can also cause an autoinflammatory syndrome characterized by excessive IL-1β secretion (MIM: 611762). Like *NLRP3*, both gain-of-function and loss-of-function variants have been described that cause increased inflammasome activation and loss of NF-κB inhibition, respectively (18, 82, 83). NLRP12-associated disorders are very rare and reported symptoms primarily include inflammation in skin and bone. Neuroinflammatory manifestations such as headache and sensorineural hearing loss are occasionally observed (17) and rare reports of optic neuritis exist (18). In addition, loss-of-function mutations in *IL1RN*, encoding the IL-1 receptor antagonist, also cause excess IL-1β signaling. These patients primarily have autoinflammatory manifestations in skin and bone but a patient with CNS vasculitis has also been reported (84).

### NF-κB Dysregulation

NF-κB constitute a ubiquitously expressed family of transcription factors that have been conserved throughout eukaryotes and that serve as key mediators of inflammatory responses (85). Upon activation, IKK phosphorylates the NF-κB inhibitor IκBα at two N-terminal serines, thereby triggering ubiquitin-dependent, proteasome-mediated IκBα degradation, resulting in rapid and transient nuclear translocation of canonical NF-κB members that can induce transcription of a variety of pro-inflammatory cytokine genes. Low penetrance variants in *TNFAIP3*, encoding A20, have been associated with many different complex immune mediated diseases (86), but more recently, heterozygous loss-of-function variants have established an autoinflammatory disease entity termed A20 haploinsufficiency (MIM: 616744), with similarities to Bechet’s disease and autoimmune lymphoproliferative syndrome (ALPS) (23, 87, 88). A20 is a ubiquitin editing enzyme and a potent inhibitor of NF-κB (89). Most cells express baseline amounts of A20 but expression is upregulated in pro-inflammatory settings (86). Deficiency of A20 consequently results in insufficient suppression of NF-κB and dysregulated innate immunity *via* overproduction of cytokines such as TNF, IL-1β, IL-18, and IL-6 (24). Sporadic reports exist of A20 haploinsufficiency with neurological symptoms, such as aseptic meningitis, and CNS vasculitis (23, 24). Furthermore, a frameshift variant in *TNFAIP3* has been reported as the cause of neuropsychiatric SLE manifested as headache, seizures, cognitive impairment, ptosis, and difficulty with upward gaze (22). The pathogenesis may involve an influence on blood brain barrier (BBB) permeability from the disease associated variant (22). Although *Tnfaip3* ^-/-^ mice are prone to fulminant neuroinflammation involving the NLRP3 inflammasome (90), the neurological spectrum in human *TNFAIP3* haploinsufficiency suggests that species differences exist or that one *TNFAIP3* allele is sufficient to restrain inflammation in the CNS.

ITCH, RNF11, and TAX1BP1 comprise additional essential components of the ubiquitin-editing protein complex that normally ensures the transient nature of inflammatory NF-κB-mediated signaling pathways (85). Autosomal recessive mutations in *ITCH* are associated with syndromic multisystem autoimmune disease (MIM: 613385) (91). Although ITCH is highly expressed in the brain, neurological symptoms are not a prominent feature of patients with ITCH deficiency. Altogether, excessive NF-κB activation is associated with autoinflammation and a low incidence of neuroinflammation. Furthermore, loss-of-function variants in several additional components of the NF-κB signaling pathway have been described with immunodeficiency, but where neuroinflammation is not a prominent feature.

### Comment on Neuroinflammation in IL-1β-Mediated Autoinflammatory Disorders

Headache is the most common neurological manifestation in the IL-1β-mediated autoinflammatory disorders. This is consistent with the emerging role for inflammasomes in migraine (92) and the association of *MEFV* variants with the risk of migraine (93). Moreover, seizures is another commonly reported neurological manifestation of these diseases. Importantly, both acute and chronic neuroinflammation has been linked to epileptogenesis (94) and in experimental systems TNF-blockade has reduced epileptic activity (95). Interestingly, the frequency of seizures in FMF patients (4%) appears to be higher than in the other disorders. This may be a consequence of unique aspects of the pyrin inflammasome such as sensing of bacterial modification/inactivation of Rho GTPases (96).

Pathogenic insights can be gained not only from studying cellular processes but also from clinical observations of what drugs have efficacy treating specific inborn errors of immunity. Most autoinflammatory diseases have been reported to respond to anti-IL-1 therapy (97). Moreover, first-line treatment for FMF is colchicine, which likely acts by interfering with the cytoskeletal changes required for inflammasome assembly, also implicating IL-1 (98). Colchicine-resistance is observed in approximately 10% of all FMF patients, which generally receive anti-IL-1 therapy as second-line treatment (99). Anti-IL-6 and anti-IL-18 therapy are occasionally used for autoinflammatory diseases in general (98). Anti-TNF therapy has been mainly used for TRAPS with inconsistent results. Anti-TNF may even provoke an inflammatory flair in TRAPS, akin to experience from MS (100, 101). A20 haploinsufficiency frequently responds to colchicine but a very wide range of treatments have been used in reported cases (102). Interestingly, experience from A20 haploinsufficiency suggests that patients who carry the same pathogenic mutation do not necessarily respond to the same treatment implying a role for genetic background and/or environment (102).

**Figure 2:**
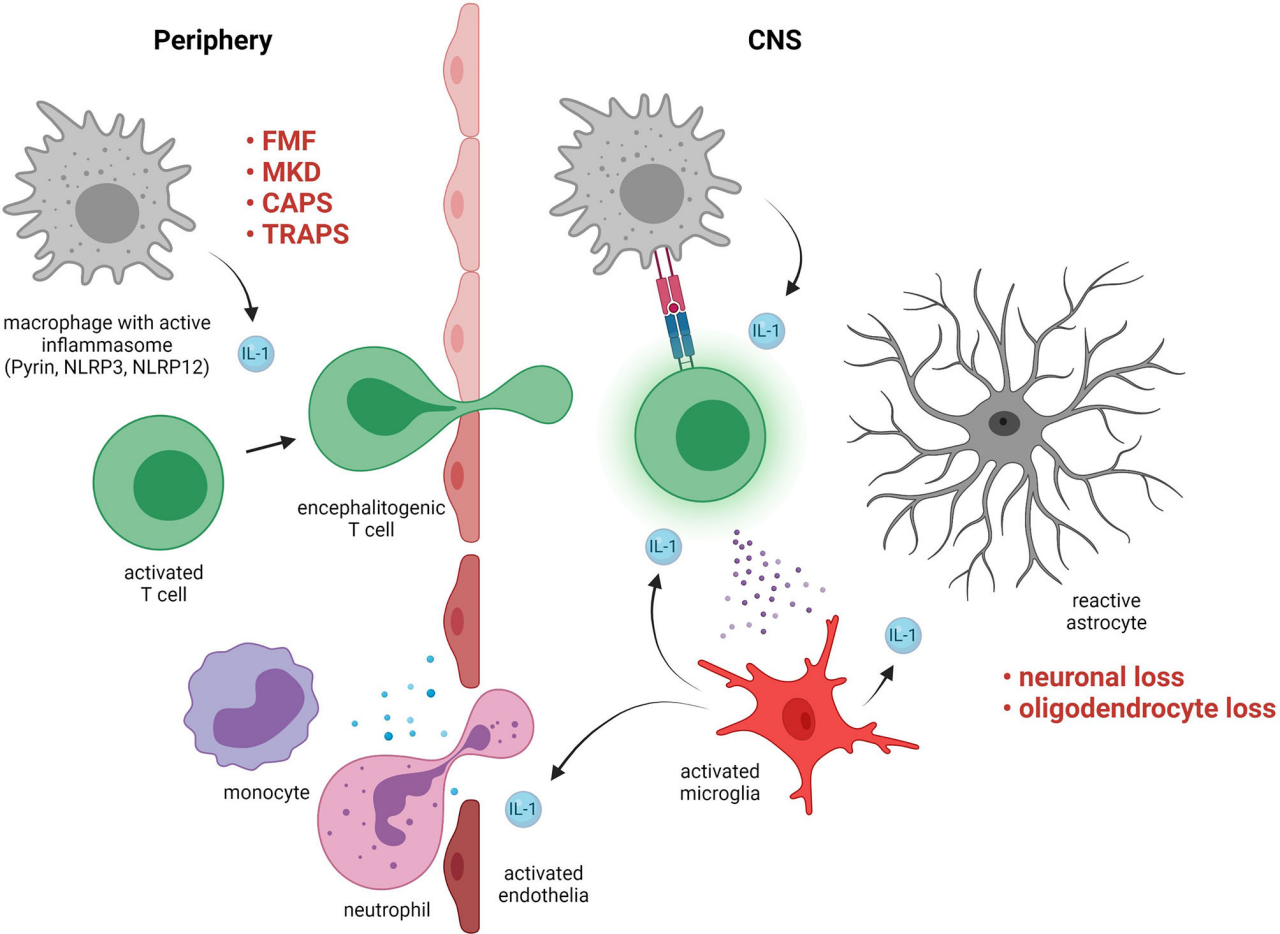
Pathogenic roles of interleukin (IL)-1-signaling in neuroinflammation. Examples of disorders with exaggerated IL-1-signaling include familial Mediterranean fever (FMF), mevalonate kinase deficiency (MKD), cryopyrin-associated periodic syndrome (CAPS), and TNF receptor-associated periodic syndrome (TRAPS). IL-1 in the periphery contributes to skewing of activated T cells towards an encephalitogenic phenotype. The T cells cross the blood brain barrier and in the central nervous system (CNS) they are activated by resident antigen presenting cells *via* MHC interactions and cytokines. The T cells activate microglia, which produce IL-1 that triggers a pro-inflammatory response in astrocytes and activates endothelia resulting in further immune cell infiltration. Maladaptive CNS-inflammation can cause irreversible damage such as loss of neurons and oligodendrocytes.

Most cells produce IL-1β to some degree but during neuroinflammation major contributors are T cells, infiltrating myeloid cells, and microglia (**Figure 2**) (103). Physiological IL-1 signaling in the CNS has a role in, for example, memory performance or neuronal survival (104) but elevated IL-1 signaling has a wide range of pathological effects (103). Which cell types in the CNS that respond to IL-1β is controversial, but sequential deletion of the IL-1 receptor in mouse CNS cells suggests that endothelial cells in the BBB as well as in cerebral ventricles are the major targets (105, 106). Microglia are activated by IL-1β *in vivo* but this may be an indirect effect *via* signaling from endothelial cells (105). For sterile neuroinflammation in general, the inciting factor that triggers inflammation is rarely understood. An inflammatory cascade that starts in the periphery and then migrates across the BBB is often hypothesized (107). In the case of IL-1β-mediated autoinflammation, a wide range of pathogen associated molecular patterns (PAMP) and damage associated molecular patterns (DAMP) can elicit IL-1β secretion with insufficient regulatory feedback mechanisms for resolution. IL-1β promotes the differentiation of IL-17 and GM-CSF-secreting T helper cells, which are implicated in the pathogenesis of MS as well as other autoimmune diseases and can cross the BBB (108–110). Once inflammation has spread to the CNS, a vicious cycle can potentially be established including IL-1β-mediated immune cell chemotaxis to the CNS *via* activated endothelial cells, skewing of T helper cells and astrocytes towards encephalitogenic phenotypes (111), as well as neuronal excitotoxicity and subsequent synaptopathy (**Figure 2**) (112).

The wide range of observed neurological presentations is intriguing considering the common pathogenesis of dysregulated inflammasomes and may reflect differences in genetic background, comorbidities, or environmental exposures. Furthermore, apart from constitutional neurological symptoms such as headache, the neuroinflammatory manifestations reported in association with IL-1β-associated autoinflammatory diseases are rare and could potentially reflect separate disease entities with complex etiology for which the autoinflammatory disease gene variants act as risk-factors. Interestingly, several of the autoinflammation associated variants are commonly implicated as modifyers of complex diseases. Bechet’s disease has similarities with FMF regarding both symptomology and treatment, which in 3-30% of cases is complicated by neuroinflammation (113). Patients with neuro-Bechet’s disease and the closely related neuro-Sweet’s disease that carry mutations in *MEFV* have more pronounced neurological manifestations such as headache and neuroimaging findings such as white matter lesions and non-parenchymal lesions (114, 115). Notably, co-occurrence of MS in carriers of homozygous or heterozygous pathogenic *MEFV* variants is well described (8, 116–118). Moreover, *NLRP12* has recently been reported as a candidate gene for familial MS (119) and *NLRP3* as well as *TNFRSF1A* variants appear to modify susceptibility and/or severity of MS (118). Thus, autoinflammatory susceptibilities can likely fuel other neuroinflammatory processes, triggering or exacerbating disease.

### Type I Interferonopathies

#### Aicardi-Goutières Syndrome

A subset of the autoinflammatory disorders called type I interferonopathies is distinguished by excess IFNα/β signaling (25). In 1984, Jean Aicardi and Françoise Goutières described a form of severe familial progressive encephalopathy in children (120). A majority of these patients have signs of upregulated type I interferon signaling, often assessed using a panel of interferon-stimulated genes as a proxy measure (121). To date, variants in at least 9 genes have been identified, which together underlie most cases of Aicardi–Goutières syndrome (AGS): *TREX1* (MIM: 225750), *RNASEH2A* (MIM: 610333), *RNASEH2B* (MIM: 610181), *RNASEH2C* (MIM: 610329), *SAMHD1* (MIM: 612952), *ADAR1* (MIM: 615010), *IFIH1* (MIM: 615846), *LSM11* (MIM: 619486), and *RNU7*-*1* (MIM: 619487) (122–127). Loss-of-function variants in these genes cause autosomal recessive disease inheritance, except for *IFIH1* and *TREX1*. Gain-of-function variants in *IFIH1* are associated with autosomal dominant inheritance, whereas for *TREX1* both recessive and dominant inheritance has been reported. Disease mechanisms include accumulation or modification of endogenous nucleic acids, enhanced activity of nucleic acid sensor or other components in the interferon signaling pathway triggering excessive type I IFN production, and loss of negative regulation (128). *TREX1* encodes an exonuclease that cleaves nucleic acids in the cytosol, thereby preventing their accumulation and triggering of a type I interferon associated inflammatory response (129), which is toxic to neurons (130). Cardinal features of AGS are early onset of a severe neurological disorder with intracranial calcification, leukoencephalopathy, cerebral atrophy, cerebrospinal pleocytosis, as well as cutaneous manifestations. As more data have been gathered, a wider range of neurological and other clinical phenotypes have been associated with *TREX1* variants (26, 131). Phenotype-genotype associations exist to some extent in AGS and regarding the neurological manifestations, variants in *TREX1* appear more prone to have an encephalitic presentation (25). Furthermore, *ADAR1* variants have been linked to the pediatric neurodegenerative disorder bilateral striatal necrosis (BSN) (132). BSN is clinically characterized by acute or subacute onset of dystonia and radiological symmetrical abnormalities in the striatum. Onset can be as late as in adolescence, often with an infectious trigger. Although clinically distinct from AGS, BSN patients with *ADAR1* variants have signs of interferonopathy such as upregulated interferon-stimulated genes and may have brain calcifications and signs of neuroinflammation such pleocytosis, elevated neopterin in CSF, and spinal cord involvement (132, 133). In summary, excessive type I interferon signaling frequently causes neuropathology but the phenotypic heterogeneity associated with these genetic variants suggests that unrecognized modifying factors have a significant influence on individual outcome.

#### Pseudo-TORCH

An early-onset syndrome caused by severe congenital infections goes under the acronym TORCH, which stands for Toxoplasmosis, Other agents, Rubella, Cytomegalovirus, and Herpes simplex. The term pseudo-TORCH is used for the same clinical picture when no infection is detected. Three genetically defined forms of pseudo-TORCH exist to date caused by recessive variants in *OCLN* (MIM: 251290), *USP18* (MIM: 617397), and *STAT2* (MIM: 618886), respectively, of which the latter two are type I interferonopathies. All three disorders cause brain malformations, intracranial calcifications, and severe developmental delay. Most reported cases have died at an early age from cerebral hemorrhage. AGS is a common differential diagnosis. Features that are more consistent with pseudo-TORCH are microcephaly, thrombocytopenia, liver dysfunction, and hepatosplenomegaly. *OCLN* codes for occludin, a tight junction protein. Occludin deficiency may cause BBB dysfunction but the pathophysiology of this disease remains unclear (134). The clinical features suggest elevated type I interferon signaling but this has not been demonstrated. USP18 is a STAT2-dependent negative regulator of type I interferon signaling and lack of USP18 or a STAT2 variant that is unable to interact with USP18 consequently result in prolonged signaling downstream of the type I interferon receptor (135–137). In the initial report of STAT2 variant pseudo-TORCH, a severe multisystemic inflammatory disorder developed at the age of 8 months, which included marked neuroinflammation in the form of brain calcifications as well as white matter and cerebellar abnormalities (137). Pseudo-TORCH therefore encompasses genetic diagnoses with considerable clinical overlap with AGS and that may present with neuroinflammation.

#### Vasculopathies

Pathologic type I interferon signaling is associated with cerebrovascular disease, both in monogenic interferonopathies and typically more complex genetic diseases such as SLE (138, 139). In addition to AGS, *TREX1* variants have been associated with the adult onset disorder retinovasculopathy and cerebral leukodystrophy with systemic features (RVCLS, MIM: 192315) (140). However, unlike AGS, *TREX1* variants associated with RVCLS do not disrupt exonuclease activity but rather manifest as gain-of-function variants with dominant inheritance. The underlying pathology of RVCLS can be vasculitis, thrombotic microangiopathy, or aneurysmal dilatation and occurs in both small and large vessels (141, 142). Moreover, *TREX1* variants have been suggested as the genetic basis of patients with cerebral small vessel disease with cerebral autosomal dominant arteriopathy with subcortical infarcts and leukoencephalopathy (CADASIL)-like phenotype (143) and monogenic SLE.

Gain-of-function variants in *STING1* cause the type I interferonopathy STING-associated vasculopathy with onset in infancy (SAVI, MIM: 615934) (144, 145). Core manifestations of SAVI are early-onset systemic inflammation, skin vasculopathy, and interstitial lung disease. Consistent with other interferonopathies cerebral calcifications are observed as a sign of CNS involvement. Occasionally CSF pleocytosis or overt neurological symptoms such as spastic paresis and seizures have been reported (27, 146).

#### Other Interferonopathies

Autosomal recessive loss-of-function variants in *ISG15* cause a syndrome termed ISG15-deficiency (MIM: 616126)^103^. ISG15 is an intracellular ubiquitin-like protein induced by type I interferon that in humans stabilizes USP18 in a feedback inhibition loop. ISG15-deficiency consequently leads to an interferonopathy but results in a milder neurological phenotype compared to pseudo-TORCH (28, 147). Moreover, ISG15 acts as an extracellular inducer of type II interferon and its deficiency is consequently also associated with increased susceptibility to mycobacterial disease. All described ISG15-deficient patients have cerebral calcifications, a sign of elevated type I interferon signaling, and seizures have frequently been reported. DNase II deficiency underlies a distinct clinical syndrome of autoinflammation and elevated type I interferon signaling. Neurological manifestations include cerebral calcifications and white matter hyperintensities (29). Loss-of- function variants in *ACP5* has been identified as a cause of spondyloenchondrodysplasia with immune dysregulation (SPENCDI, MIM: 607944), a rare autosomal recessive syndrome characterized by skeletal and immune abnormalities (30, 148). *ACP5* encodes an enzyme that regulates the activity of osteopontin and is involved in non-nucleic acid dependent type I interferon induction. A wide spectrum of immune dysregulation associated with SPENCDI has been described, including development of SLE. Neurological manifestations include developmental delay, spastic paresis, and intracranial calcifications (149). Mutations in *PSMB8* is a cause of a disorder previously called chronic atypical neutrophilic dermatosis with lipodystrophy and elevated temperature (CANDLE). The disease now belongs to a subcategory of interferonopathies called proteasome-related autoinflammation (PRAAS, MIM: 256040) and is called PRAAS1. Like other interferonopathies cerebral calcifications have been reported but also aseptic meningitis (31, 150, 151). Thus, disease-causing variants in an increasing spectrum of genes are being linked to interferonopathies with neuroinflammatory manifestations.

### Comment on Neuroinflammation in Type I Interferonopathies

Unlike the IL-1β-mediated autoinflammatory diseases where conspicuous neuroinflammation is rare, most interferonopathies have signs of CNS involvement. Assuming that the intracranial calcifications observed in virtually all interferonopathies are caused by type I interferon, this can either be a result of direct interferon signaling in cells of the brain or of signals transduced from CNS cells that are in contact with the peripheral circulation. Notably, in homeostatic conditions, neither IFN-α nor IFN-β readily cross the BBB and reach the brain parenchyma (152–154) but as the barrier opens in inflammatory settings, cytokines and cells can gain access to the CNS. Although all somatic cells can produce type I interferons, the major contributors are the plasmacytoid dendritic cells (DC) (155, 156). In a mouse model of AGS, *Trex1* deletion restricted to the hematopoietic compartment was sufficient for full disease, whereas deletion restricted to DCs yielded less pronounced manifestations (157). On the other hand, transfer of wild type bone marrow into irradiated *Trex1* ^-/-^ mice did not rescue inflammation, implying that non-hematopoietic cells also are sufficient to trigger the type I IFN-mediated pathology (158). In inflammatory settings, CNS cells that produce type I interferon include microglia, astrocytes, and neurons. During homeostasis, microglia present in white matter receive a constant interferon signal, which may be necessary to increase housekeeping functions like phagocytosis of myelin debris (159). Furthermore, constitutive interferon signaling is required for the homeostasis of neurons in mice demonstrated by the development of age associated neurodegeneration with similarities to Parkinson’s disease and Lewy body dementia in *Ifnb* ^–/–^ mice, with a likely contribution from defective autophagy (160). Humans that lack type I interferon signaling are rare and reported examples of *IFNAR1* and *IFNAR2* deficiencies have all been in children (161–163) or young adults not specifically assessed for signs of neurodegenerative disease (164).

In inflammatory settings, most CNS cells including infiltrating immune cells have the potential to respond to type I interferon (165). In a mouse model of MS, USP18 expression specifically in microglia has an important role in dampening excessive interferon input that otherwise would result in pathological microglia activation, which reflects the human interferonopathy caused by USP18 deficiency (135). In contrast, IFN-β is a well-established therapy for MS (166) and has been reported to reduce the severity of mouse models of the disease (167). Notably, not all MS patients respond to IFN-β, and exacerbations have been described (168, 169). Moreover, NMO, as well as several autoimmune diseases that do not primarily affect the CNS, are likely worsened by type I interferon and it has been hypothesized that IFN-β is an effective treatment in Th1-biased but not in Th17-biased diseases or disease subphenotypes (170). Although it is not definitively established that type I interferons are driving the disease manifestations of the interferonopathies it is at least likely. AGS and MS are immunologically and clinically very different and the proposed T helper cell polarizing effect of IFN-β in MS is probably not a dominant feature in the type I interferonopathies.

Signal transduction downstream of the type I interferon receptor involves activation of JAK1 and TYK2 followed by activation of STAT1 and STAT2, which together with IRF9 translocate to the nucleus to initiate transcription of interferon stimulated genes (ISG). Increasing experience suggests that treatment with JAK inhibitors is efficacious in some patients with interferonopathies (171). However, in one study, treatment with JAK inhibition before onset of symptoms did not prevent development of AGS (172). Additionally, based on the hypothesis that endogenous retroelements drive interferon signaling in AGS, a small (n=8) open-label pilot study of reverse transcriptase inhibitor therapy has been conducted with promising results (173).

In summary, interferon-related autoinflammation is qualitatively different from the classical autoinflammatory disorders with prominent IL-1β-biased inflammation. The neuroinflammation observed in interferonopathies is likely less dependent on recruitment of innate or adaptive immune cells. Although the pathogenesis is clearly related to excess type I interferon signaling, what part of this complex system is appropriate to target pharmacologically for these disorders is not well understood.

### Hemophagocytic Syndromes

#### Familial Hemophagocytic Lymphohistiocytosis

Hemophagocytic lymphohistiocytosis (HLH) is a rare hyperinflammatory syndrome characterized by fever, cytopenias, and splenomegaly, which often leads to a sepsis-like condition with multiple organ dysfunction. HLH can be divided into familial HLH (FHL) caused typically by recessive mutations and secondary non-Mendelian forms. FHL is most frequently caused by loss-of-function variants in genes required for lymphocyte cytotoxicity, including genes that are required for CD8^+^ T cell and NK cell exocytosis. To date, mutations in *PRF1* (MIM: 603553), *UNC13D* (MIM: 608898), *STX11* (MIM: 603552), *STXBP2* (MIM: 613101), and *RHOG* have been linked to FHL (174–178). Both FHL and secondary HLH often have an apparent triggering event, for example an infection or malignancy, leading to uncontrolled immune stimulation. The defective killing of target cells makes the host unable to clear the antigenic stimulus and control activated immune cells. This results in persistence and amplification of the immune response with excessive secretion of pro-inflammatory cytokines. In turn, the pro-inflammatory cytokines activate macrophages and mediate much of the tissue damage. Diagnostic HLH criteria have been established and a diagnosis is made based on the fulfillment of 5/8 of the criteria (179). Neuroinflammatory manifestations occur at any time during the disease course in FHL or secondary HLH but are not part of the diagnostic criteria. In pediatric HLH, neurological symptoms or signs have been reported in 13-73% of patients and pathological MRI or CSF findings in 33-91% (32). Importantly, when the disease presents as an isolated neuroinflammatory disorder, perhaps without the diagnostic criterion cytopenia, HLH may not be included as a differential diagnosis (180, 181). An international survey and literature review of confirmed FHL cases that presented as an isolated neuroinflammatory disease (CNS-HLH) has been reported (33). Common symptoms at presentation in this case series were ataxia, seizures, headache, motor impairment, and visual abnormalities. Brain MRI findings were non-specific and included a range of multifocal bilateral abnormalities including demyelination in the hemispheres, basal ganglia, cerebellum, or brain stem. Occasionally MRI may reveal a single mass-like lesion (182) or may mimic septic embolization (183). MRI abnormalities have been reported in FHL due to *PRF1* variants even in the absence of neurological symptoms (184). Therefore, CNS-HLH is frequently initially diagnosed as acute demyelinating encephalomyelitis (ADEM), CNS vasculitis, meningitis, leukodystrophy, or MS (38, 185–188). The mean time from onset of symptoms of CNS-HLH to confirmed molecular diagnosis is more than two years in published cases and it is recognized that a fully developed HLH disease can arise several years after onset of CNS-HLH (33). Two patients with cerebellar involvement on MRI and consistent symptoms in the form of ataxia and dysarthria were shown to have had a FHL-associated biallelic *PRF1* mutations without developing FHL before dying at the age of 1 and 5 years, respectively (189). Conversely, when in remission and off treatment for typical FHL, a relapse may present as isolated CNS-HLH (190). Importantly, compared to most of the other disorders reviewed herein, FHL is prone to rapidly develop into a life-threatening inflammatory condition and vigilance is required regarding early symptoms, which may be from the CNS.

#### Familial Hemophagocytic Lymphohistiocytosis-Related Disorders

An additional set of inherited disorders also frequently cause HLH. These include Griscelli syndrome type 2 (GS2, MIM: 607624), Chediak-Higashi syndrome (CHS, MIM: 214500), X-linked lymphoproliferative syndrome (XLP) type 1 (XLP1; MIM: 308240) and XLP type 2 (XLP2; MIM: 300635).

A distinct feature of GS2 and CHS is hypopigmentation because of defective granule transport in melanocytes. GS2 is an autosomal recessive disease caused by loss-of-function variants in *RAB27A*, which encodes a protein that facilitates secretory lysosome trafficking and exocytosis in several cell types (191). The impaired cytotoxic activity in T and NK cells can result in increased susceptibility to infections as well as failure to control immune responses and development of HLH. Microglial dependence on Rab27a for migration towards sites of injury in the CNS suggests a possible mechanism for neuroinflammation in the setting of Rab27a deficiency (192). A GS2 patient with isolated CNS-HLH presenting with developmental regression, seizures, and eventually status epilepticus has been described (193). Other cases of GS2 have been reported with neurological involvement in the form of encephalopathy, headache and tonsillar herniation, focal seizures, multifocal or diffuse white matter lesions, and pathological CSF (38–40). An atypical case of GS2 without hypopigmentation describes a 14-year old male that presented with myoclonus, dysmetria, dysarthria, ataxia, fever, and pancytopenia (37). The neuroinflammatory manifestations resolved within 3 months despite conservative management but returned after 2 months with hemiparesis, abducens nerve palsy, fever, and pancytopenia. Chediak-Higashi syndrome may have neurological manifestations, but these are almost always neurodegenerative, and MRI typically reveal atrophy but no focal lesions (194, 195).

Loss-of-function variants in *SH2D1A* and *XIAP* cause XLP type 1 and 2, respectively, both with X-linked recessive inheritance. *SH2D1A* codes for an adaptor protein expressed in T cells but its role in the pathogenesis of XLP1 is not fully established (196). Deletion of *SH2D1A* may result in mild immunodeficiency early in life in males but can also present as early-onset HLH. Clinical manifestations of XLP1 are typically triggered by Epstein-Barr virus (EBV) infection, resulting in deficient humoral immunity, polyclonal proliferation of B and T cells, lymphoma, and HLH. A case of Burkitt lymphoma in a 14-year old male with hypogammaglobulinemia followed by fatal CNS vasculitis has been reported (41). The CNS vasculitis manifested as short-term memory deficit and MRI revealed multiple edematous and hemorrhagic lesions. The symptomatology of other reported cases of XLP and CNS vasculitis includes headache, blurred vision, seizures, memory impairment, motor deficits. CSF analysis may reveal elevated white cells and protein. MRI often show aneurysms, T2 hyperintensities, and hemorrhagic lesions (42–45). Thus, severe neuroinflammatory symptoms are not isolated to FHL patients with defective lymphocyte cytotoxicity.

### Comment on Neuroinflammation in Hemophagocytic Syndromes

Although all characterized gene variants that underlie FHL result in impaired lymphocyte cytotoxicity, the tendency for CNS involvement in FHL subtypes likely differ. Neuroinflammation in FHL due to *PRF1* variants was observed in 36% of 124 cases in a multi-center study (197). The corresponding proportion associated with *UNC13D* variants was estimated at 60% (198, 199) and with *STXBP2* variants at approximately 50% (34). Estimates were less certain for other FHL types but a study suggests that CNS involvement is less frequent in FHL due to *STX11* variants (200). Furthermore, it is becoming increasingly recognized that hypomorphic variants with some residual function, often cause FHL with later onset and atypical clinical presentations, for example isolated CNS involvement (201–206).

The immunopathogenesis of HLH is described as a cytokine storm. Several genetic defects that predispose to HLH impair the delivery of cytotoxic granules from CD8^+^ T cells and NK cells, which is essential both for clearance of pathogens but also of activated antigen presenting cells. A feed-forward loop is initiated with increasing tissue infiltration and activation of immune cells that secrete a plethora of pro-inflammatory cytokines, most notably IFN-γ, TNF, IL-1, IL-2, IL-6, IL-10, IL-12, IL-18, and GM-CSF. Consequently, the established treatments for HLH involve broad immunosuppression to achieve remission while hematopoietic stem cell transplantation is needed to cure primary HLH (207). Animal models of FHL imply that IFN-γ derived from CD8^+^ T cells drive the pathogenesis (208, 209). However, HLH-like disease in the absence of IFN-γ has also been reported recently (210). There is a large body of work on the role of IFN-γ in neuroinflammation. Although protective effects have been suggested, most data point towards a pathogenic role. An early trial in MS patients showed that administration of IFN-γ worsened disease (211). Briefly, in experimental conditions, low doses of IFN-γ generally exert protective effects on neurons, microglia and oligodendrocytes whereas high doses appear to have the opposite effect (211). Although neutralization of specific cytokines such as TNF and IL-1 have been used as second line treatment for HLH, their efficacy is unknown. Novel treatment strategies that are being pursued include anti-IFN-γ and JAK-inhibition (212, 213).

In summary, the CNS is vulnerable to the hyperinflammation associated with FHL and related disorders, but even severe cases can have a good outcome if diagnosed early so that appropriate treatment can be initiated in parallel with arrangements for a hematopoietic stem cell transplantation.

## Tolerance Defects

Loss-of-function variants in *FOXP3*, a key transcription factor in the development of regulatory T (Treg) cells, is the cause of X-linked recessive immunodysregulation polyendocrinopathy enteropathy X-linked (IPEX, MIM: 304790). The Treg cell defect leads to loss of peripheral tolerance manifested as enteropathy, dermatitis, type I diabetes mellitus, hypoparathyroidism, autoimmune cytopenias, and other autoimmune diseases. IPEX-like syndromes may also arise due to defects in other Treg cell related genes such as loss-of-function variants in *IL2RA2*, *CTLA4*, and *LRBA*, or gain-of-function variants in *STAT1* and *STAT3 (* 214). A cohort of 173 patients with IPEX or IPEX-like syndrome have been reported and approximately 25% in both groups had neurological symptoms, which included seizures, ventriculomegaly, and developmental delay (46).

ALPS (MIM: 601859) is an autosomal dominant disease characterized by abnormal lymphocyte survival resulting in lymphoproliferation, autoimmunity, and secondary malignancies. Loss-of-function variants in Fas is the most common cause followed by FasL, and caspase-10. Autoimmunity typically manifests as autoimmune cytopenia but essentially any organ can be affected including the nervous system (47). ALPS without a specified genetic basis has been associated with NMO and Guillain Barré syndrome (47, 48).

Loss-of-function variants in *CTLA4*, a negative regulator of T cell activation has been described as a cause of ALPS (ALPS type V, MIM: 616100) but has more recently been defined as a disorder of its own. *CTLA4* haploinsufficiency may cause a range of autoimmune and lymphoproliferative manifestations. In a series of 133 patients with CTLA-4 insufficiency, a penetrance of 67% was observed and upper range of age of onset was 50 years (215). Among the affected, 29% had neurological features. A wide range of neurological syndromes were observed including autoimmune encephalitis, demyelination, increased intracranial pressure, ischemic or hemorrhagic lesions. Others have described cerebellar lesions and spinal cord degeneration (49, 50). Altogether, neuroinflammatory manifestations are thus a feature in up to a third of patients with defects in immune tolerance.

## Immunodeficiency Disorders

The complement system consists of approximately 30 different components and deficiencies have been described for all of them. The clinical consequences depend on which component is affected, but the spectrum broadly involves susceptibility to infections, autoimmunity, cardiovascular disease. Loss-of-function variants in any of the three subcomponent genes for C1q, i.e. *C1QA*, *C1QB*, or *C1QC*, cause the autosomal recessive condition C1q-deficiency (MIM: 613652), which stands out among the complement component deficiencies for being strongly linked to development of SLE. One study found that neurological symptoms at disease presentation occur to a similar extent in C1q-deficient and C1q-sufficient SLE/SLE-like patients (216) but C1q-deficient patients with SLE appear to relatively often present with neuropsychiatric symptoms (51). Seizures are a common manifestation of neuropsychiatric-SLE but encephalopathy, infarcts, and transverse myelitis have also been described (217). Neuroimaging findings include calcifications or ischemic lesions in the basal ganglia, vasculitis, and brain atrophy. Intracranial calcifications, a cardinal feature of the interferonopathies, allude to increased type I interferon also having an influence on the CNS in this SLE subset, possibly explained by the observed inhibitory role of C1q on type I interferon induction (52, 53, 218). C1q is expressed by neurons and opsonizes unwanted synapses for removal by microglia (219). Plausible indirect mechanisms for the observed neurological involvement include C1q-dependent removal of apoptotic cells or immune complexes. Most complement deficiencies are associated with increased susceptibility to bacterial meningitis due to the important role of complement for the elimination of encapsulated bacteria, such as meningococci (220). Aseptic meningitis has also been associated with several of the complement deficiencies, which often manifests as neutrophilic inflammation, making the distinction from septic meningitis difficult (51, 221–223).

Variants in *TLR3* as well as *TRIF*, *TRAF3*, *TBK1*, *IRF3*, and *NEMO* that code for mediators downstream of TLR3, and *UNC93B1* that is involved in TLR3 trafficking, all reduce type I interferon signaling and are reported genetic causes of herpes simplex encephalitis (HSE) (224–229). Disease penetrance is incomplete and very few HSE multiplex families have been described. Interestingly, even complete absence of TLR3 signaling does not appear to increase susceptibility to other infections than herpes simplex (230). Autoimmune encephalitis relatively often occurs after HSE, likely due to bystander activation and breaking of tolerance in the CNS (231). Thus, one can speculate that the risk of CNS autoimmunity is elevated in patients with type I interferon deficiencies.

Heterozygous loss-of-function variants in *GATA2* (MIM: 614172) cause a wide range of defects including cytopenias, particularly affecting monocytes, B cells and NK cells, resulting in susceptibility to infections in addition to a risk of developing myelodysplastic syndrome/acute myeloid leukemia (232). GATA2 is an essential transcription factor for hematopoietic stem/progenitor cell renewal and differentiation (233). In a survey with 79 patients, the median age of onset was early adulthood and 8% were symptom-free at the age of 40 (54). Three of these patients (4%) presented with neurological symptoms in the form of intellectual disability, transient ischemic cerebral palsy, and progressive multifocal leukoencephalopathy (PML), respectively. PML is an often fatal neuroinflammatory consequence of reactivation of latent JC-virus, which is observed in patients with immunodeficiency, primary or secondary to a wide range of immunosuppressive drugs (234).

COPA encodes a protein involved in endoplasmic reticulum (ER)-Golgi transport. Variants in the gene cause the rare COPA-syndrome (MIM: 616414), mostly associated with manifestations in the lungs, kidneys, and joints. However, a case of COPA-syndrome and NMO has been described (235). The patient had bacterial meningitis at the age of 2 and onset of NMO at the age of 6. Immunological findings associated with loss of function COPA-variants include Th17 polarization and humoral autoimmunity consistent with the immunopathology of NMO (236, 237).

Loss-of-function variants in *RANBP2* are associated with the autosomal dominant post-infectious and often fatal complication acute necrotizing encephalopathy type 1 (ANE1, MIM: 608033) (238, 239). Many different viruses and some bacteria have been implicated but no association appears to exist to severity of ANE1, suggesting that genetic or other factors are more influential after the initial trigger. *RANBP2* encodes the nuclear pore protein RAN binding protein 2 that is ubiquitously expressed and has several described cellular functions. Hypothesized pathogenic mechanisms include metabolic and mitochondrial dysfunction as well as induction of a cytokine storm. TNF and IL-6 are elevated in the serum and CSF of ANE1 patients, but elevated cells in the CSF is not typically observed (239). Taken together, the rarity of these immunodeficiency disorders makes a causal relationship to any observed CNS involvement difficult to establish but for several of them there are plausible neuropathological mechanisms that lend some support to the presence of etiological links.

### Comment on Neuroinflammation in Tolerance Defects and Immunodeficiency Disorders

Several of the neuroinflammatory manifestations described in these sections result from immune deficiencies leading to defective tolerance mechanisms and autoimmunity. IPEX and ALPS are prototypical autoimmune syndromes caused by defective Treg cell formation and lymphocyte apoptosis, respectively. The complement system contributes to clearance of potential autoantigens and type I interferon defects lead to fulminant infections, possibly resulting in bystander activation of autoreactive lymphocytes. However, these and other autoimmune syndromes are primarily associated with involvement of other organs than the CNS. Similarly, the observation that autoimmune diseases tend to cluster in families does not apply to CNS autoimmunity such as MS to the same extent (240). Together this implies that distinct requirements are needed for tolerance in the CNS to break. Although CNS surveillance of adaptive immune cells is well-described the threshold of activation is higher inside the BBB (241). Moreover, activation of innate immune cells locally by PAMPs or DAMPs does not elicit infiltration of neutrophils or monocytes, as is the case in most other tissues (242).

The mechanisms that underlie neuroinflammation in COPA or RANBP2 deficiency are unknown. These genes are expressed in most tissues throughout the body including the CNS (gtexportal.org) and therefore a neuroinflammatory cascade that originates within the CNS may be hypothesized, in contrast to most other gene defects described herein that clearly have major direct consequences on immune cells, thus making an inflammatory reaction that is initiated in the periphery and then spills over to the CNS a more likely scenario.

In brief, the pathogenic mechanisms that underlie neuroinflammation in disorders of immune tolerance or defense are elusive and likely heterogeneous. There is not much evidence that supports the involvement of actual autoreactive encephalitogenic lymphocytes in neuroinflammation related to tolerance defect disorders or any other disease category reviewed herein. New single cell methodologies can potentially shed light on pathophysiologic mechanisms underlying these sets of immune disorders.

## Conclusion

With the growing use of high-throughput exome and genome sequencing in clinical practice, it has become increasingly recognized that adult onset of monogenic disease is not necessarily uncommon (243). Moreover, genetic variants associated with early and late onset Mendelian diseases may not be the same and adjustment of filtering criteria may be needed when analyzing the sequencing data (244). As genome sequencing is increasingly applied for clinical diagnostics, more disease-causing variants will be identified. The task of filtering through the large number of variants that likely have no clinical significance will continue to be a challenge. One important consideration when assessing the possible causative role of disease-associated variants is whether they are consistent with the patient’s phenotype. Herein we have summarized reported neuroinflammatory phenotypes observed in association with monogenic diseases. These CNS-manifestations are often not part of the typical disease description and therefore relevant pathogenic variants may be overlooked when tasked with matching sequencing results with a clinical neuroinflammatory presentation.

Based on available reports, some conclusions can be drawn regarding the distinguishing features of neuroinflammation in relation to the underlying immunopathogenesis. For example: IL-1β driven autoinflammation is often associated with headache and to a lesser extent seizures and aseptic meningitis, interferonopathies with chronic cerebral abnormalities such as calcifications and developmental defects, and HLH with prominent but unspecific signs neuroinflammation on MRI and in CSF. As awareness increases regarding the potential CNS involvement in inborn errors of immunity, the clinical characterization of reported cases will improve. This will help better define what is a typical neuroinflammatory manifestation in relation to the various types of immune defects, which in turn will facilitate the process of making a genetic diagnosis.

## Author Contributions

HL reviewed the literature and drafted the manuscript. YTB revised the manuscript. All authors contributed to the article and approved the submitted version.

## Conflict of Interest

The authors declare that the research was conducted in the absence of any commercial or financial relationships that could be construed as a potential conflict of interest.

## Publisher’s Note

All claims expressed in this article are solely those of the authors and do not necessarily represent those of their affiliated organizations, or those of the publisher, the editors and the reviewers. Any product that may be evaluated in this article, or claim that may be made by its manufacturer, is not guaranteed or endorsed by the publisher.

## Glossary

**Table:**

MS	multiple sclerosis
NMO	neuromyelitis optica
CNS	central nervous system
MRI	magnetic resonance imaging
CSF	cerebrospinal fluid
FMF	familial Mediterranean fever
CAPS	cryopyrin-associated periodic syndrome
MKD	mevalonate kinase deficiency
MVA	mevalonic aciduria
HIDS	Hyperimmunoglobulinaemia D syndrome
TRAPS	TNFR-associated periodic syndrome
PAAND	Pyrin-Associated Autoinflammation with Neutrophilic Dermatosis
FCAS	familial cold autoinflammatory syndrome
MWS	Muckle-Wells syndrome
CINCA syndrome	chronic infantile neurological cutaneous and articular syndrome
ALPS	Autoimmune lymphoproliferative syndrome
BBB	blood brain barrier
PAMP	pathogen-associated molecular pattern
DAMP	damage-associated molecular pattern
AGS	Aicardi-Goutières syndrome
BSN	bilateral striatal necrosis
TORCH	Toxoplasma gondii, other agents, rubella, cytomegalovirus
RVCLS	Retinovasculopathy and cerebral leukodystrophy with systemic features
CADASIL	Cerebral autosomal dominant arteriopathy with subcortical infarcts and leukoencephalopathy
SAVI	STING-associated vasculopathy with onset in infancy
SPENCDI	Spondyloenchondrodysplasia with immune dysregulation
CANDLE	Chronic Atypical Neutrophilic Dermatosis with Lipodystrophy and Elevated Temperature
PRAAS	proteasome-associated autoinflammatory syndrome
DC	dendritic cell
ISG	interferon-stimulated gene
HLH	Hemophagocytic lymphohistiocytosis
FHL	familial haemophagocytic lymphohistiocytosis
ADEM	Acute disseminated encephalomyelitis
GS2	Griscelli syndrome type 2
CHS	Chediak-Higashi syndrome
XLP	X-linked lymphoproliferative syndrome
EBV	Epstein-Barr virus
Treg	regulatory T cell
IPEX	Immunodysregulation polyendocrinopathy enteropathy X-linked syndrome
HSE	Herpes simplex encephalitis
PML	Progressive multifocal leukoencephalopathy
ER	endoplasmic reticulum
ANE1	Acute necrotizing encephalopathy type 1
